# Rhenium *N-*heterocyclic carbene complexes block growth of aggressive cancers by inhibiting FGFR- and SRC-mediated signalling

**DOI:** 10.1186/s13046-020-01777-7

**Published:** 2020-12-07

**Authors:** Alice Domenichini, Ilaria Casari, Peter V. Simpson, Nima Maheshkumar Desai, Lingfeng Chen, Christopher Dustin, Jeanne S. Edmands, Albert van der Vliet, Moosa Mohammadi, Massimiliano Massi, Marco Falasca

**Affiliations:** 1 Metabolic Signalling Group, School of Pharmacy & Biomedical Sciences, Curtin University, Perth, WA 6102 Australia; 2 Curtin Institute of Functional Molecules and Interfaces, Department of Chemistry, Curtin University, Perth, WA 6102 Australia; 3 Department of Biochemistry & Molecular Pharmacology, New York University School of Medicine, New York, NY 10016 USA; 4 Department of Pathology and Laboratory Medicine, Larner College of Medicine, University of Vermont, Burlington, VT 05405 USA; 5 Curtin Health Innovation Research Institute, Curtin University, Perth, WA 6102 Australia

**Keywords:** Rhenium complexes, Pancreatic cancer, Neuroblastoma, FGFR, Src

## Abstract

**Background:**

Platinum-based anticancer drugs have been at the frontline of cancer therapy for the last 40 years, and are used in more than half of all treatments for different cancer types. However, they are not universally effective, and patients often suffer severe side effects because of their lack of cellular selectivity. There is therefore a compelling need to investigate the anticancer activity of alternative metal complexes. Here we describe the potential anticancer activity of rhenium-based complexes with preclinical efficacy in different types of solid malignancies.

**Methods:**

Kinase profile assay of rhenium complexes. Toxicology studies using zebrafish. Analysis of the growth of pancreatic cancer cell line-derived xenografts generated in zebrafish and in mice upon exposure to rhenium compounds.

**Results:**

We describe rhenium complexes which block cancer proliferation in vitro by inhibiting the signalling cascade induced by FGFR and Src. Initially, we tested the toxicity of rhenium complexes in vivo using a zebrafish model and identified one compound that displays anticancer activity with low toxicity even in the high micromolar range. Notably, the rhenium complex has anticancer activity in very aggressive cancers such as pancreatic ductal adenocarcinoma and neuroblastoma. We demonstrate the potential efficacy of this complex via a significant reduction in cancer growth in mouse xenografts.

**Conclusions:**

Our findings provide a basis for the development of rhenium-based chemotherapy agents with enhanced selectivity and limited side effects compared to standard platinum-based drugs.

## Background

Cisplatin has been a frontline treatment for many cancers over the last 40 years [1, 2], and is still used for cancers of the bladder, ovary and testis, as well as for cancers of the head and neck. Although cisplatin has been successfully used in the treatment of many cancer types, the fact that it functions via random DNA targeting and induces harsh side effects, such as neurotoxicity and nephrotoxicity, makes it a far from ideal therapeutic agent [3]. The development of chemoresistance is also a significant limiting factor in the use of this drug [2, 4]. Carboplatin, another platinum-based drug that directly interferes with DNA metabolism, leads to the formation of cross-links in a manner similar to cisplatin. However, its more stable structure confers the benefit of having fewer side effects [5, 6], although its impact on bone marrow results in a reduction in blood cells including platelets [7]. A further platinum-based drug with cytotoxic activity is oxaliplatin, which differs mechanistically from cisplatin and carboplatin as it promotes ribosome biogenesis stress leading to cell death [2]. Because of the severe side effects that result from the use of all platinum-based drugs, other metal compounds are being studied for their anti-proliferative activity, with the goal of discovering new agents that are better tolerated by cancer patients.

Rhenium is a transition metal that has been recently studied for the development of novel anticancer agents [8–10]. Its anticancer activity was first observed with tricarbonyl bismine complexes in HeLa cells [1, 4, 11–13]. Several different complexes have been designed which utilize a combination of organometallic ligands, such as N- or S-based ligands, oxo groups or peptides [14]. Rhenium-containing compounds have been found to have promising anticancer activity associated with apoptosis [15], necrosis [16], autophagy [17], mitophagy [18] and oxidative stress [19]. Notably, rhenium-based compounds have a great potential as theranostics [20]. Indeed, rhenium possesses two isotopes, ^186^Re and ^188^Re, that have been used in nuclear medicine for more than fifty years [21]. Rhenium use in medicinal pharmaceuticals, radio-imaging and luminescent probes has led to its consideration for use in diagnostic therapy. For example, Rhenium-188 hydroxyethylidenediphonate (^188^Re-HEDP) is a drug that has been repurposed for the use in the treatment of prostate cancer with promising outcomes [22]. In addition, when taxanes, known to increase the sensitivity of tumour cells to radiotherapy, and Rhenium compounds were combined for the treatment of prostate cancer, a significant additive anti-proliferative activity was observed [23]. In spite of increasing evidence for their antiproliferative activity and the fact that different rhenium-based compounds have a much lower toxicity compared to other heavy metals, their mechanisms of action and structure activity relationships are not well understood, with only a few studies published to date [1, 9, 24].

Over the past few years, we have focused on the anticancer properties of rhenium tricarbonyl complexes bound to bidentate *N*-heterocyclic carbine ligands rather than di-imine ligands (Re-NHC) [10, 25]. These compounds have demonstrated antiproliferative activity in pancreatic, neuroblastoma and ovarian cancer cells, while possessing low toxicity. Here we present an investigation of the mechanism of action of Re-NHC complexes and the results of experiments to test their activity in vivo.

## Methods

### Cell culture

Human pancreatic cancer and neuroblastoma cell lines were obtained from the American Type Culture Collection (ATCC) and cultured as per ATCC® guidelines. AsPC-1 (ATCC® CRL-1682™) and BxPC3 (ATCC® CRL-1687™) required RPMI-1640 as base medium, with the addition of foetal bovine serum (FBS) to a final concentration of 10% and glutamine to a final concentration of 2 mM. HPAF-II (ATCC® CRL-1997™) cells were cultured in Eagle’s Minimum Essential Medium (EMEM) with the addition of FBS to a final concentration of 10% and glutamine to a final concentration of 2 mM. SW1990 (ATCC® CRL-2172™) were cultured in Leibovitz’s L-15 medium, with the addition of FBS to a final concentration of 10%. SH-SY5Y (ATCC® CRL-2266™) cells were grown in Dulbecco’s modified Eagle’s Medium (DMEM) supplemented with 10% FBS, 1% penicillin/streptomycin, 2 mM glutamine and 10 mM sodium pyruvate. IMR-32 (ATCC® CCL-127™) cells were cultured in RPMI-1640 medium containing 10% FBS, 2 mM glutamine, 10 mM sodium pyruvate and 10 mM non-essential amino acids (NEAA).

Pancreatic cancer tumorspheres enriched in cancer stem cells were cultured according to the protocol previously described by Domenichini et al. [26]. Primary mouse pancreatic cancer cells KPC were used according to the protocol previously described [26]. The KPC (KrasLSL.G12D/+; p53R172H/+; PdxCretg/+) mouse model is a clinically relevant genetically engineered mouse model (GEMM) for PDAC. Mutationally activated Kras and mutated p53 drive the development of a pancreatic intraepithelial neoplasia that eventually progresses into pancreatic ductal adenocarcinoma with pathological features closely resembling the human disease [27]. LAN-1, KELLY and the primary human neuroblastoma cell line (hNB) were provided by Professor Arturo Sala (Brunel University) and cultured as described [28].

### Western blots

Cell lysates and tissues were homogenised in RIPA buffer (50 mM TRIS-HCl pH 7.4, 150 mM NaCl, 1% NP-40, 0.1% SDS, 5 mM EDTA) supplemented with a protease and phosphatase inhibitor cocktail (Roche). To terminate the reaction, SDS sample buffer [125 mM Tris- HCl (pH 6.8), 6% SDS, 20% glycerol, and 0.02% bromophenol blue supplemented with 10% β-mercaptoethanol] was added and the samples boiled for 10 min. Proteins were separated on SDS-PAGE 8 to 12% Tris-glycine gels (Life Technologies) and transferred to a nitrocellulose membrane (BioRad). For protein detection, membranes were incubated in 3% BSA in TBS/0.05% Tween-20 blocking buffer for 1 h at room temperature, and incubated overnight at 4 °C with primary antibodies from Cell Signaling Technology®: Phospho-FGFR^(Y653/654)^ (#3471), Phospho-Src Family^(Y416)^ (#2101), N-Myc (#9405), Phospho-Akt^(T308)^ (#9275) and GAPDH (#5174), the latter used as a loading control. The following day, membranes were incubated with HRP-conjugated secondary antibodies (Cell Signaling Technologies) at a 1:40000 dilution in 0.75% BSA in TBS/0.05% Tween-20 buffer for 1 h at room temperature. Signal was detected using the chemiluminescent detection reagent Amersham ECL Prime Western Blotting Detection Reagent (GE Healthcare Life Sciences) and imaged using BioRad ChemiDoc XRS+.

### Soft agar Colony formation

The anchorage independent soft agar colony formation assay is a well-established protocol to measure the proliferative capacity of cancer cells [29]. Media were prepared as 2X concentrated solutions, including noble agar (Sigma-Aldrich) as two stocks to be diluted with 2X media solutions, 1.2% for the first layer and 1% for the second layer (the cell layer). Six well plates were coated with a first layer (2 mL) of 1.2% noble agar in 2X RPMI (final 0.6% agar in 1X media). Cells were counted using trypan blue exclusion and about 3 × 10^4^ cells were resuspended in 5 mL of 0.5% noble agar (1% noble agar in 2X media) prior to treatment. For each six well plate, the first row (3 wells) was seeded with cells treated with JVG045 (10 μM) and the second row with cells treated with DMSO. Once the first layer had settled, 1.5 mL of cell suspension containing approximately 1 × 10^4^ cells in 0.5% noble agar was distributed on top of the first layer. The top layer was then covered with complete RPMI and the plates incubated at 37 °C in a 5% CO_2_ incubator for 4 weeks. Media were removed and colonies fixed for 10 min at room temperature with 10% Methanol/10% glacial acetic acid. Colonies were stained with 0.05% crystal violet solution (Sigma-Aldrich) for 30 min at room temperature on a rocking shaker and subsequently washed with water on a rocking shaker to clear the agar. Colonies were imaged using BioRad ChemiDoc XRS+ and counted using ImageJ.

### Kinase profiler

A screen was done to determine the effect of JVG045 and Ps27 on the activity of a panel of protein-kinases via SelectScreen Kinase Profiling Service (Invitrogen-Life Technologies, Paisley, UK). The assay used 1 μM of various compounds and ATP at the concentration noted (see Supplementary Tables).

### Analysis of effects of JVG045 on tyrosine trans-phosphorylation activity of FGFR1^Cys488^ kinase

N-terminally His-tagged FGFR1^Cys488^ kinase carrying a native cysteine at position 488 in the glycine-rich loop was expressed in BL21 (DE3) *E. coli* cells at 20°C overnight. Cells were lysed in 25 mM HEPES pH 7.5 buffer containing 150 mM NaCl and 10% glycerol. Cell lysis and subsequent column chromatography purifications were done in presence reducing agent (i.e., 10 mM β-mercaptoethanol or 2 mM DTT) to avoid Cys488-mediated disulfide-linked dimerization of kinases. Following high speed centrifugation at 20,000 x g, supernatant was filtered, and loaded onto Ni^2+^ metal affinity chromatography column (5 mL) (GE Healthcare Life Sciences). Bound FGFR1^Cys488^ kinase was eluted with 18 column volumes of linear gradient of 0.5 M imidazole, and applied onto a SourceQ anion exchange chromatography column (20 mL) (GE Healthcare Life Sciences). Column was developed with 13 column volumes of linear gradient of 1 M NaCl. Fractions containing FGFR1^Cys488^ kinase were pooled, concentrated, and applied to a Superdex 75 column (GE Healthcare). Eluents containing kinase protein were then pooled, concentrated and incubated overnight with FastAP™ Thermosensitive Alkaline Phosphatase (#EF0651; Thermo Scientific), and re-purified by Source Q column chromatography as above to obtain highly homogenous phosphorylation-free FGFR1^Cys488^ kinase.

Purified FGFR1^Cys488^ kinase was incubated with or without compound JVG045 (100 μM) overnight at 4°C. *Trans*-phosphorylation on tyrosines was initiated by mixing FGFR1^Cys488^ kinase with a reaction buffer containing ATP and MgCl_2_ to final concentrations of 67.5 μM (kinase), 25 mM (ATP) and 50 mM MgCl_2_. Reactions were quenched at different times (as indicated in figure) by adding EDTA (final concentration, 50 mM) to the reaction mixture. The progress of FGFR kinase *trans*-phosphorylation was monitored by native-PAGE (#17062401, GE Healthcare).

### Src kinase assay

Recombinant Src was expressed in BL21-AI *Escherichia coli* (Thermo) containing the pEX-Src-C-His (Origene, Blue Heron Biotech) and purified in a modified procedure as previously described [30]. The Src kinase assay used was the ADP-GLO assay (Promega) according to manufacturer protocol. Specifically, the kinase (1 ng) was incubated with between 10 nm-10 mM JVG045 compound/AZD0530 in 15 μL of kinase buffer for 30 min prior to the addition of 10 μL substrate solution containing ATP and poly[4Glu:Tyr] (Sigma). This reaction was allowed to react at RT for 1 h prior to quenching with ADP-Glo reagent for 40 min, followed by the addition of ADP-GLO detection reagent for 30 min prior to reading luminescence on a 96-well microplate reader.

### Animal experiments

#### Toxicology studies

24 h post fertilisation (hpf), zebrafish embryos were equally distributed into wells of a 24-well plate (about 10 embryos/well) and treated with increasing concentration of selected compounds as shown in Supplementary Fig. 2. Compounds were prepared in DMSO and then diluted to the final concentration in embryo medium (E2), which is a physiological solution [31].

Embryos were observed daily at 24 h intervals and toxicity scores (hatching and mortality) were recorded until 120hpf. For heart rate assessments, embryos were anesthetized with tricaine (ethyl 3-aminobenzoate methanesulfonate) at 48hpf and counted under the stereomicroscope for 1min. To assess JVG045 teratogenicity compared to BGJ398, embryos were treated with the compounds starting from 2hpf and results observed at 24hpf. Compounds were prepared in DMSO and diluted to the final concentration in E2 embryo medium. DMSO was used as negative control.

#### Zebrafish Xenografts

For zebrafish xenografts, wild type Tübingen (TU) zebrafish were bred and maintained in the Western Australian Zebrafish Experimental Research Centre (Biomedical Research Facility- Shenton Park, Western Australia). Experiments and data analyses were done as previously described [32]. Briefly, HPAF-II human pancreatic cancer cells were incubated with Vibrant™-Dil dye (ThermoFisher Scientific) 4 μL/mL in HBSS at 37 °C for 10 min, followed by 15 min on ice in the dark. Cells were then harvested and resuspended at a density of 10^7^ cells/, loaded into a capillary glass needle using a puller (p-97 Flaming/Brown by Sutter Instrument®) and 10 nL of cell suspension (approximately 100 cells/embryo) was injected in the perivitelline space of 24-h post fertilisation (hpf) zebrafish embryos. Zebrafish were incubated at 34 °C O/N to allow for cell growth and the following day embryos were equally distributed in to three treatment groups. One group did not receive any treatment or cells (blank), a second group was treated with 0.1% DMSO and a third group was treated with 10 μM JVG045. At 5 days post fertilisation (dpf), the effect of the drugs on cancer cell growth was documented using a fluorescent stereomicroscope equipped with a digital camera (Nikon SMZ Zoom). Images were analysed using ImageJ. Non injected embryos were used to subtract background fluorescence.

### Mice Xenografts

Six to seven-week-old NOD/SCID (NOD.CB17-Prkdcscid/Arc) immune-deficient mice were purchased from the Animal Resources Centre (ARC-Murdoch-Western Australia) and maintained under pathogen-free conditions with water and food provided ad libitum. Mice were injected subcutaneously randomly on either the left or the right flank with 3.5 × 10^6^ HPAF-II human pancreatic cancer cells following a previously described protocol [32]. When tumours reached a volume of about 50 mm^3^ (according to the formula: tumour volume = 1/2(length × width^2^), mice were randomized into two groups and treated with either vehicle (0.5% carboxymethyl cellulose (CMC)/0.4% Tween-80) or JVG045 30 mg/kg as a daily intra-peritoneal injection (IP) in a volume of 250 μL. Animal health conditions were monitored daily and tumours were measured three times a week by an unbiased operator until the largest tumours reached a volume of 1500mm^3^. Procedures involving animals and their care were established according to the institutional guidelines in compliance with national and international policies (Curtin Animal Ethics Committee Approval 2016–40).

### Reactive oxygen species (ROS) measurement by flow Cytometry

The increase in production of reactive oxygen species (ROS) was measured using the oxidation of 2′-7′ dichlorofluorescin diacetate (H2DCF-DA Sigma Aldrich D6883). Cells were seeded in a 6-well plate at a density of 3.5 × 10^5^ cells/well and incubated overnight. The following day, cells were treated with either DMSO or JVG045 (10 μM) for one hour. Antimycin (50 μM) was used as positive control. Cells were then washed with PBS and loaded with 1 μg/mL of H2DCF-DA diluted in serum-free medium for one hour while protected from light. After incubation, cells were detached, washed twice with ice-cold PBS and resuspended in PBS containing 1 μg/mL propidium iodide to exclude dead cells. Corresponding unstained controls were also prepared. Cells were analysed using a BD FACSCantoTMII flow cytometer and data were analysed using FlowJo® software.

### Physiochemical and metabolic parameters

Physiochemical and metabolic parameters studies have been performed at the Centre for Drug Candidate Optimisation, Monash University, as described below.

- *Calculated physicochemical parameters using ChemAxon JChem software*
  A range of physicochemical properties were calculated using the Chem Axon chemistry cartridge via JChem for Excel software (version16.4.11).
- *Kinetic Solubility Estimation using Nephelometry (SolpH)*
  Compound in DMSO was spiked into either pH 6.5 phosphate buffer or 0.01 M HCl (approxpH2.0) with the final DMSO concentration being 1%. After 30 min had elapsed, samples were then analysed via Nephelometry to determine a solubility range [33].
- *Distribution Coefficient Estimation using Chromatography (gLogDpH)*
  Partition coefficient values (LogD) of the test compounds were estimated at pH 7.4 by correlation of their chromatographic retention properties against the characteristics of a series of standard compounds with known partition coefficient t values. The method employed is a gradient HPLC based derivation of the method developed by Lombardo et al. [34].

### In vitro metabolic stability

The metabolic stability assay was performed by incubating the compound (0.5 μM) in liver microsomes at 37 °C and a protein concentration of 0.4 mg/mL. The metabolic reaction was initiated by the addition of an NADPH-regenerating system and quenched at various timepoints over a 60-min incubation period by the addition of acetonitrile containing diazepam as internal standard. Control samples (containing no NADPH) were included (and quenched at 2, 30 and 60 min) to monitor for potential degradation in the absence of cofactor. The human liver microsomes used in this experiment were supplied by XenoTech, lot#1410230. The mouse liver microsomes used in this experiment were supplied by XenoTech, lot#1510256.

### Statistical analyses

The results presented are representative of at least three independent experiments. Statistical analyses were done using GraphPad Prism v.8.4.2, and normalisation of data was applied where appropriate. Statistical significance was considered at a value of *p* < 0.05.

## Results

### Re-NHC complexes with inhibitory activity towards FGFR1 and Src

We recently showed that Re-NHC complexes suppress the growth of pancreatic cancer cell lines by blocking the cells in the G2/M phase via a mechanism involving the inhibition of phosphorylation of aurora kinase A [25]. We have now identified a subset of compounds as good candidates for further studies and for possible therapeutic drug development. Two of these compounds, JVG045 and ps27 (see structures in Supplementary Fig. 1) showed good pharmaco-toxicological profiles and were selected for activity analysis in more detail (see below and Supplementary Fig. 2).

**Fig. 1:**
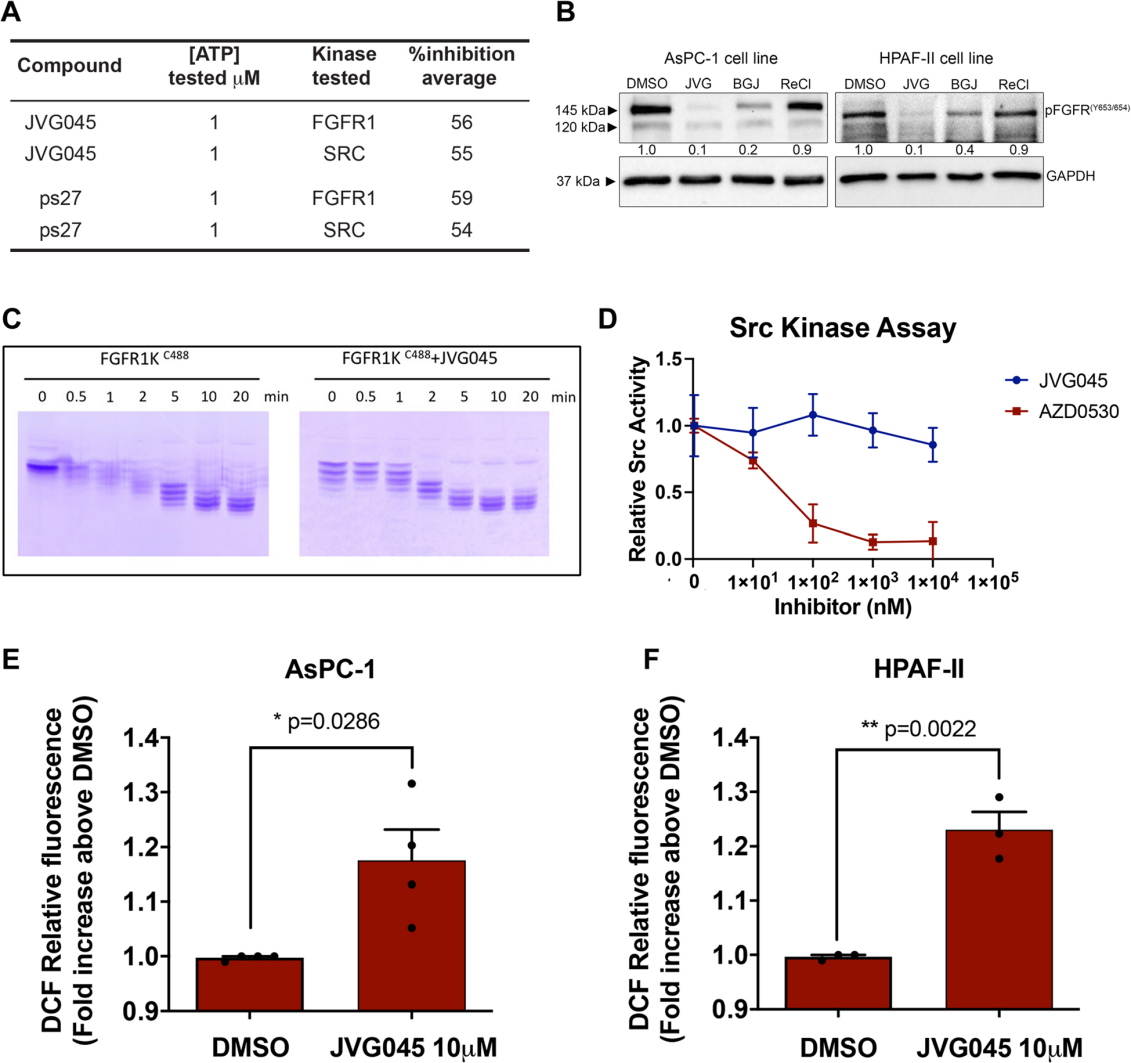
*Mechanism of action of tricarbonyl rhenium complexes.* **a** Summary of SelectScreen Kinase Profiling showing the % inhibition of JVG045 and ps27 on the kinases FGFR1 and Src. **b** Western blots in pancreatic cancer cell lines AsPC-1 and HPAF-II showing that JVG045 (10 μM) indeed inhibits the phosphorylation of FGFR1 with an effect comparable to the selective FGFR inhibitor BGJ398 (5 μM); ReCl was used as negative control. **c** Autophosphorylation activity of FGFR1, no difference in kinase activity is detected in the presence of JVG045, indicating that there is no direct inhibition. **d** Src kinase assay shows that, when used on the purified kinase, JVG045 does not have a direct action compared to the selective Src inhibitor AZD0530. **e**, **f** JVG045 (10 μM) increases the production of reactive oxygen species (ROS) in pancreatic cancer cell lines AsPC-1 (**e**) and HPAF-II (**f**) measured using the H2DCF-DA probe. Results are expressed as Mean ± SEM and are representative of at least three independent experiments. Mean fluorescence intensity DMSO: AsPC1 = 5368 + 1517 (*n* = 4); HPAF-II =4094 + 1454 (*n* = 3)

To gain insight into their mechanism of action, we did a large unbiased cell-free assay involving a protein kinase screen using these compounds (SelectScreen Kinase Profiling Service, Invitrogen-Life Technologies). This screen involves a single point inhibition assay at 1 μM against more than 120 kinases (Supplementary Table 1). Compounds JVG045 and ps27 showed > 50% inhibitory activity towards Fibroblast Growth Factor Receptor (FGFR1) and Src (Fig. 1a) and did not inhibit (percentage of inhibition < 40%) any of the other kinases tested. Further evidence for this inhibition was obtained by Western blot analyses (Fig. 1b), which were used to compare the levels of phosphorylated Fibroblast Growth Factor Receptor (pFGFR) in two different pancreatic cancer cell lines (HPAF-II and AsPC-1) using glyceraldehyde-3-phosphate dehydrogenase (GAPDH) to normalise protein content. The level of pFGFR1 Tyr653/654 was analysed under normal conditions (i.e., DMSO control) and after incubation with JVG045 at 10 μM. We focussed on JVG045 rather than ps27 because of the latter’s toxicity, as explained further below. The effect of JVG045 was also compared with an equal amount of a known specific FGFR inhibitor, BGJ398, as a positive control; the inert [Re(CO)3(phen)Cl] (where phen is 1,10-phenanthroline; the complex is referred herein to as ReCl) compound was used as a negative control (Supplementary Fig. 1). The results clearly show that JVG045 reduces the levels of phosphorylation of FGFR with an effect comparable to BGJ398. Furthermore, we assessed the effect of JVG045 on FGF-induced downstream signalling and we found that JVG045 strongly attenuated FGF-induced Akt phosphorylation at Thr308 in both AsPC-1 and HPAF-II (Supplementary Fig. 3).

Surprisingly, assessment of the substrate phosphorylation activity of purified FGFR1 showed no difference in kinase activity in the presence of 10 μM JVG045 (Fig. 1c). Furthermore, a direct kinase assay for Src using JVG045 at various concentrations showed no difference in protein activity compared to the specific Src inhibitor Saracatinib (AZD0530, Fig. 1d). These results indicate that the inhibitory activity of JVG045 on FGFR1 and Src kinases is indirect, either as a kinase inhibitor or as a covalent inhibitor [35]. In addition, JVG045 was more efficient than ps27 in inhibiting the phosphorylation of FGFR in pancreatic cancer cell lines ASPC-1 (see Supplementary Fig. 4A) and HPAF-II (Supplementary Fig. 4B). We therefore set out to determine the mechanism of action of JVG045 on the phosphorylation of FGFR1 and Src.

It has been shown that both FGFR1 and Src can be inactivated by a mechanism involving the direct oxidation of a specific cysteine residue (Cys-277 in Src and Cys-488 in FGFR1) [36]. Furthermore, it has been suggested that tricarbonyl rhenium complexes exert anticancer activity by elevating intracellular levels of reactive oxygen species (ROS) [17]. Therefore, we determined the ability of JVG045 to induce the production of intracellular ROS. Our results show that, at a concentration of 10 μM, JVG045 induces a significant increase in ROS production in human pancreatic cancer cell lines AsPC-1 (*p* = 0.0286; Fig. 1e) and HPAF-II (*p =* 0,0022; Fig. 1f). We conclude that JVG045 inhibits FGFR1 and Src indirectly, possibly through oxidation of cysteine residues.

### Toxicology studies in zebrafish

**Fig. 2:**
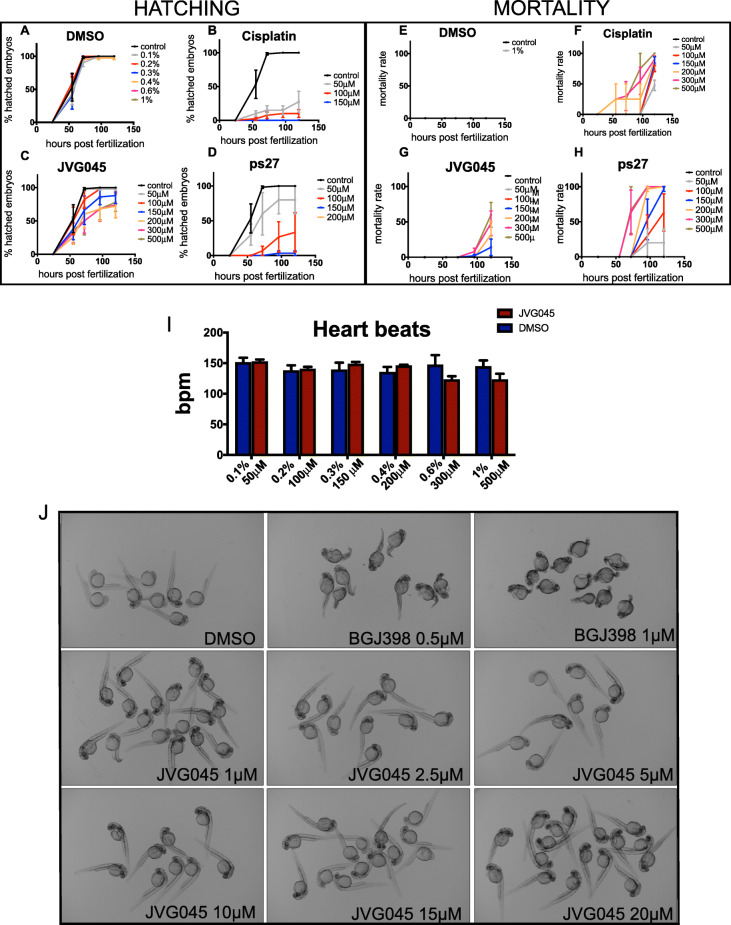
In vivo *toxicity of tricarbonyl rhenium complexes JVG045 and ps27.* (A-H) Graphs comparing the effect of a dose response of JVG045, ps27, Cisplatin and DMSO control on zebrafish hatching (**a**-**d**) and embryo mortality (**e**-**h**) from 24 h post fertilisation (hpf) until 120hpf. **i** Effect of a dose response of JVG045 on zebrafish heartbeats showing no significant difference up to 500 μM compared to DMSO. **j** BGJ398 is a competitive FGFR1–3 inhibitor [38, 39] acting on multiple FGFRs in the zebrafish embryo and causing developmental defects starting at concentrations as low as 0.5-1 μM. On the other hand, as a putative FGFR1 inhibitor, JVG045 does not cause developmental defects in the zebrafish embryo. Treatment started at 2hpf and images were taken at 24hpf. Results are showed as Mean ± SEM and are representative of at least three independent experiments

With the intent to test compounds on mouse models in vivo, we first verified their toxicity using zebrafish as a screening platform. Six compounds were selected based on previous in vitro results and compared to 1% DMSO (as a control) and Cisplatin, in a dose response experiment done by dissolving the drugs in zebrafish embryo medium in 24-well plates. Compounds JVG080, ps139a and ps197c formed crystals in the embryo medium (a physiological solution), and were therefore discarded from further investigation as unpromising drug candidates because of their limited solubility (Supplementary Fig. 2). We therefore proceeded with toxicity studies on zebrafish embryos in which we compared the toxic effect of JVG045 and ps27 to cisplatin. DMSO was used as negative control. Hatching and mortality rate are widely accepted parameters for the evaluation of substance toxicity using the zebrafish model [37]. Zebrafish embryos normally hatch around 3 days post fertilisation; our data show that when DMSO (Fig. 2a) was included in the embryo medium, at increasing concentration, 97.3% (±3.7 SD) of the embryos hatched after 3 days (72 h post fertilisation, hpf) and 99.6% (±0.9 SD) of the larvae were free from their chorion at 5 days (120hpf). On the other hand, with cisplatin, even at the lowest concentration (50 μM) only 15% (±12.9 SD) of the embryos hatched at 72hpf, increasing to an average of 27.5% (±30.9 SD) at 120hpf (Fig. 2b). In comparison with JVG045 at a concentration 10 times higher (500 μM), 50% (±46.9 SD) of the embryos hatched at 72hpf, increasing to an average of 78 (±30.3 SD) at 120hpf (Fig. 2c). In contrast, ps27 showed toxicity starting at a concentration of 100 μM, when only 6.7% (±11.5 SD) of embryos hatched after 72hpf, with an average of 33.3% (±49.3 SD) of hatched larvae after 120hpf (Fig. 2d). Mortality rate was assessed daily until 120hpf (5 days); mortality in the presence of 1% DMSO, which was null (Fig. 2e), was considered as the reference control. In the presence of cisplatin, 47.5% (±17.1 SD) of larvae died by 120hpf at 50 μM and the mortality rate reached 100% at a concentration of 500 μM, with 25% (±50.0 SD) of embryos already dying at 55hpf (Fig. 2f). However, when zebrafish embryos were exposed to JVG045 at the highest concentration (500 μM), mortality was null at 55hpf and reached 60% (±39.4 SD) at 120hpf (Fig. 2g). In comparison, when the embryos were exposed to ps27, the mortality rate at 120hpf was 63.3% (±46.2 SD) at a concentration five times lower than JVG045 (100 μM) and reached 96.7% (±5.8 SD) at 150 μM (Fig. 2h). We conclude that JVG045 has the lowest toxicity index and consequently offers significant potential as a drug candidate.

Given these encouraging data, we investigated additional toxicity parameters on zebrafish embryos by monitoring the effects of increasing concentrations of JVG045 and corresponding percentages of DMSO on heartbeat. The normal zebrafish embryonic heartbeat rate is 140–180 beats per minute (bpm); this parameter is an established criterion for the evaluation of substance toxicity [40]. Our analyses showed that heartbeat rates did not differ from data reported in the literature across all concentrations (DMSO average 142.0 bpm ± 11.17 SD; JVG045 average 138.4 bpm ± 12.95). In addition, across all concentrations, there was no significant difference in the heartbeat rate between embryos exposed to either control or JVG045 (Paired t-test t (5)=0.5706, *p* = 0.5930; Fig. 2i).

We took advantage of zebrafish transparency and rapid development, which allow easy detection of developmental defects in the main organs and structures [41], to examine the effects of JVG045 on embryogenesis. BGJ398 is a fibroblast growth factor receptor (FGFR 1–3) inhibitor [38], and when tested in zebrafish at a concentration of 0.5-1 μM, it impairs the proper development of the embryo in the posterior mesoderm and tail morphogenesis [35]. We found that JVG045 did not show any zebrafish embryonic teratogenicity, consistent with the notion that the mechanism of action of JVG045 is indirect (Fig. 2j). In addition, in zebrafish *fgfr* genes display functional redundancy, and thus inhibition of the activity of only one of these genes activates a compensatory activity from the other genes [42] such that development proceeds normally.

### JVG045 inhibits KPC mouse-derived primary cancer cell growth and reduces their anchorage-independent growth

**Fig. 3:**
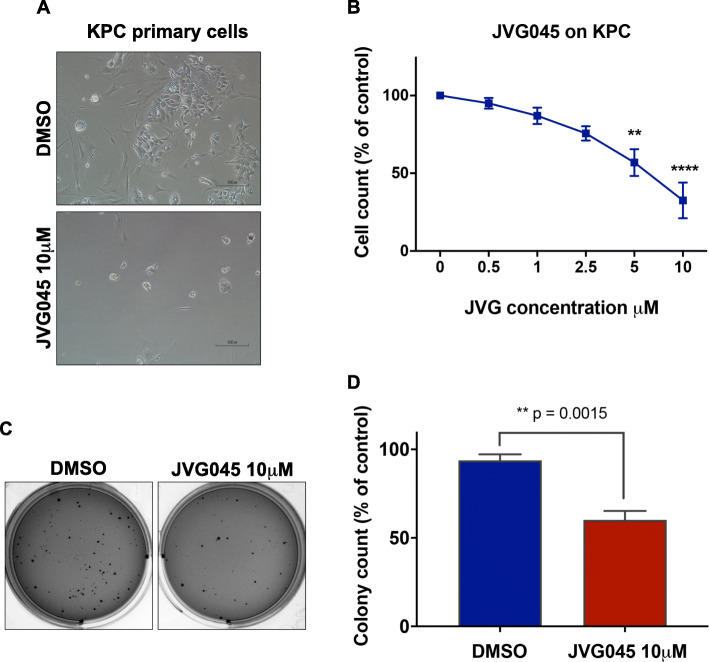
*Effect of JVG045 on primary pancreatic cancer cells from the KPC mouse model.* **a** Representative images showing the effect of JVG045 at 10 μM on the number of primary pancreatic cancer cells isolated from the KrasLSL.G12D/+; p53R172H/+; PdxCretg/+ (or KPC) mouse model, compared to DMSO. **b** Graph showing a dose response effect of JVG045 on KPC cells, with 5 and 10 μM being the most effective concentrations. **c** Representative mages showing the effect of JVG045 (10 μM) on anchorage-independent colony formation of KPC cells, compared to DMSO. **d** Graph showing a significant reduction in KPC colonies formed in the presence of JVG045 (10 μM), compared to DMSO. Experiments were performed in triplicate and showed as Mean ± SEM

To better understand the potential efficacy of the selected Rhenium compound in a tumour setting, we tested the effect of JVG045 on primary cell cultures derived from pancreatic ductal adenocarcinoma isolated from KrasLSL.G12D/+; p53R172H/+; PdxCretg/+ (or KPC) mice. We found that JVG045 impairs KPC primary cell growth in a dose-dependent manner (Fig. 3a, b), reaching statistical significance at 5 and 10 μM (One-way ANOVA F (4,20)=11.87, *p* < 0.0001). We also tested the effect of 10 μM JVG045 on anchorage independent soft agar colony formation, which measures the ability of cancer cells to grow and to proliferate without support on a solid surface [29, 43]. Our data showed that at this concentration, JVG045 significantly impaired the anchorage-independent growth of KPC primary cell colonies in soft agar (two-sample t-test, t _(6)_  =  5.544; *p* = 0.0015; Fig. 3c, d).

### Re-NHC complexes show anticancer activity in neuroblastoma cell lines with an amplified MYCN oncogene

**Fig. 4:**
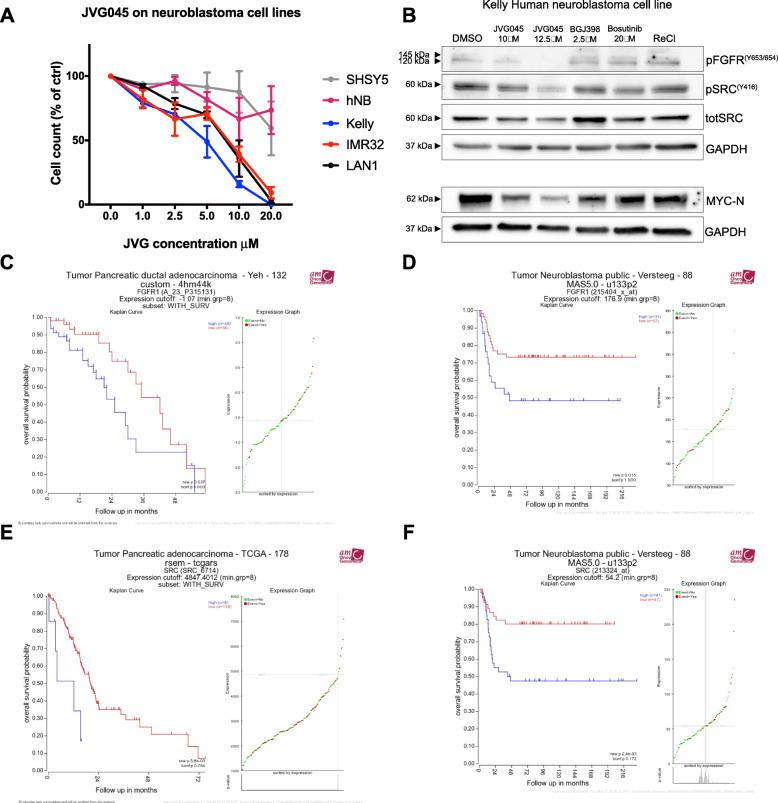
*Effect of JVG045 on human neuroblastoma cell lines.* **a** Dose response of JVG045 on human neuroblastoma cell lines. The graph shows that the tricarbonyl Rhenium compound has very little effect on cell number in those cell lines with non-amplified *MYCN* (SHSY5 and hNB), while it has a significant effect in *MYCN-*amplified cell lines (Kelly, IMR-32 and LAN-1). **b** Representative western blot showing the effect of JVG045 (10 μM) on the phosphorylation of FGFR1(Y653/654) and Src(Y416) compared to the FGFR inhibitor BGJ398 and the Src inhibitor Bosutinib, respectively. ReCl was used as a negative control. Data from R2: Genomics Analysis and Visualization Platform (http://r2.amc.nl) databases illustrating the prognostic value of FGFR1 and Src in Pancreatic Ductal Adenocarcinoma (PDAC) (**a** and **e**), and Neuroblastoma (**d** and **f**). Results are showed as Mean ± SEM and experiments were performed in triplicate

To determine the efficacy of JVG045 on different cell types, we screened a variety of cell lines derived from various cancer types (Table 1). These experiments showed that this compound is particularly active in inhibiting in vitro growth of human neuroblastoma cell lines containing an amplification of the *MYCN* proto-oncogene (Kelly, IMR-32, LAN-1), while having only a limited effect on neuroblastoma cell lines lacking *MYCN* amplification (Fig. 4a) [44]. For example, treatment with JVG045 at up to 20 μM in the *MYCN*-non-amplified cell line SHSY5 had no significant effect on cell number (One-way ANOVA, F _(5, 9)_=1.157, *p* = 0.3989). Similarly, in the *MYCN*-non-amplified primary human neuroblastoma cell line hNB, treatment with JVG045 up to 20 μM had no significant effect (F _(5, 9)_=1.649, *p* = 0.2421). In contrast, in the *MYCN*-amplified cell lines Kelly (F _(5, 12)_=51.56, *p* < 0.0001) and IMR-32 (F _(5, 12)_=21.37, *p <* 0.0001), the effect of JVG045 treatment on cell number reduction was significant starting at 2.5 μM (*p* = 0.0084 in Kelly and *p* = 0.0212 in IMR-32). JVG045 also significantly ablated cell number in another *MYCN*-amplified cell line, LAN-1 (One-way ANOVA, F _(5, 12)_=36.06, *p <* 0.0001) starting at a concentration of 5 μM (*p* = 0.0192). Figure 4b shows a representative Western blot analysis showing the Kelly human neuroblastoma cell lines treated with JVG045, together with the FGFR inhibitor BGJ398 and the Src kinase inhibitor Bosutinib. Data from the R2: Genomics Analysis and Visualization Platform (http://r2.amc.nl) databases confirm that FGFR1 (Fig. 4c, d) and Src (Fig. 4e, f) are unfavourable prognostic markers for pancreatic cancer adenocarcinoma and human neuroblastoma.

**Table 1:**
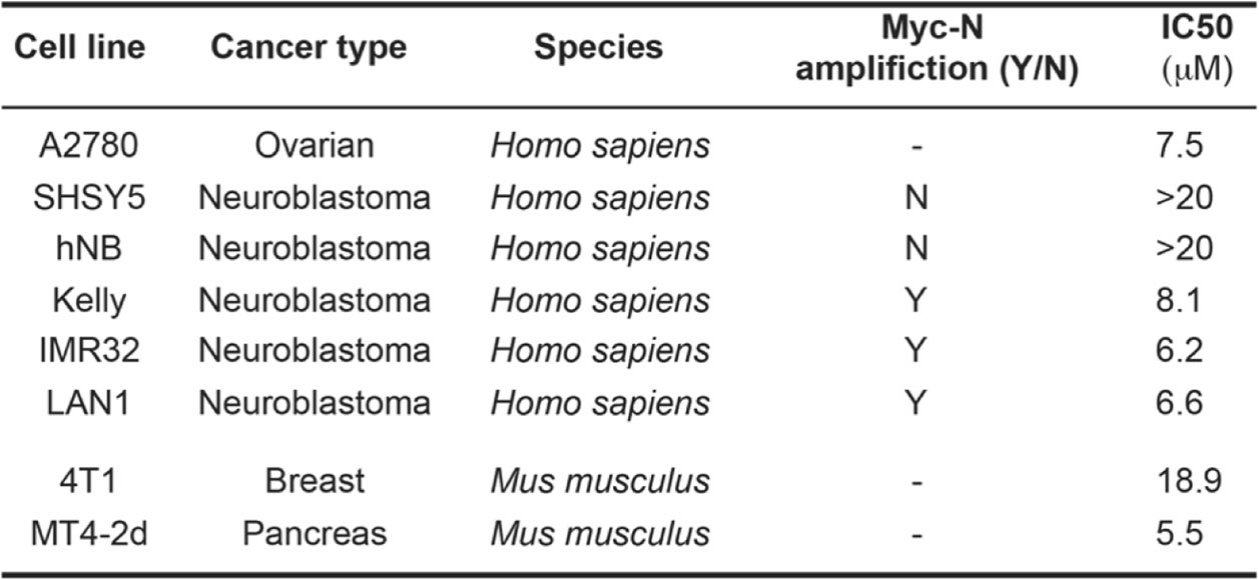
*JVG045 IC50 values for different human and murine cancer cell lines*

### Oncogene KRAS status determines responsiveness to JVG045 in pancreatic cancer cell lines

**Fig. 5:**
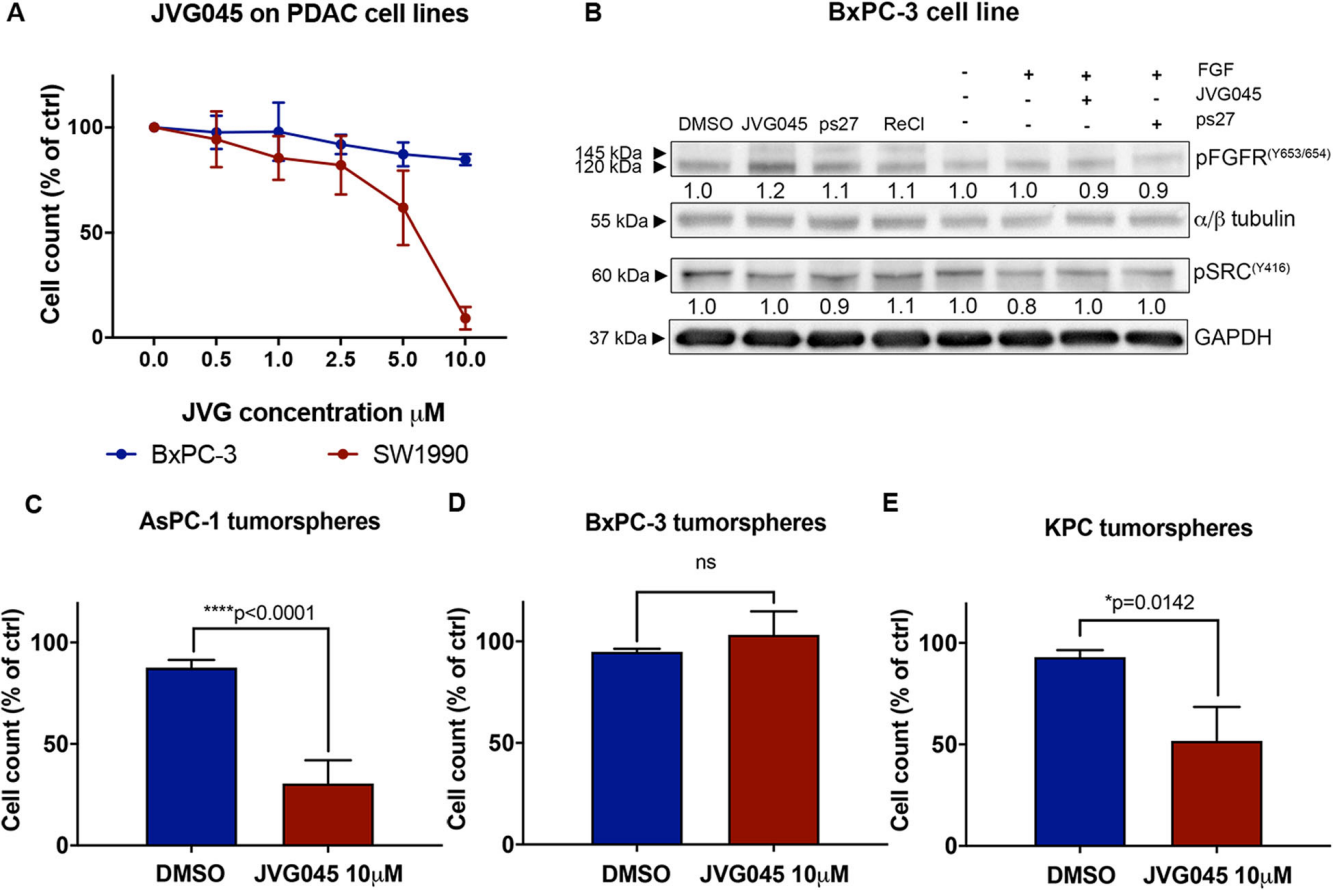
*Effect of JVG045 on pancreatic cancer cell lines depends on mutationally activated KRAS.* **a** Graph showing that in the BxPC-3 human pancreatic cancer cell line (which lacks a *KRAS* mutation) increasing doses of JVG045 had no significant effect on cell number. In contrast, SW1990, a pancreatic cancer cell line bearing a *KRAS* mutation and wt *P53,* showed enhanced sensitivity to increasing concentrations of JVG045. **b** Representative western blot showing the effect of JVG045 and ps27 (both at 10 μM) on the phosphorylation of FGFR1(Y653/654) and Src(Y416) in the presence or absence of the FGF ligand (20 ng/ml). ReCl was used as a negative control. **c** Effect of JVG045 (10 μM) in significantly reducing cell numbers in chemoresistant AsPC-1 tumorspheres enriched in cancer stem cells. **d** JVG045 (10 μM) shows no significant effect in *KRAS* wt BxPC-3 tumorspheres. **e** Effect of JVG045 (10 μM) in significantly reducing cell number in chemoresistant primary KPC tumorspheres enriched in cancer stem cells. Results are expressed as Mean ± SEM and are representative of at least three independent experiments

To test the effect of JVG045 on pancreatic cancer cells with varying degrees of genetic complexity (ATCC® TCP-1026), we exposed a range of cell lines to JVG045. These experiments showed that, compared to K-RAS mutated cell lines (IC50 AsPC-1 4.0 ± 1.2 μM, HPAF-II 5.6 ± 0.6 μM, CFPAC-1 5.7 ± 2.8 μM [25] and Supplementary Table 2), JVG045 had an insignificant effect on the only cell line that contains wild-type RAS and is not RAS-activated, BxPC-3 (IC50 > 20 μM; One-way ANOVA, F _(5, 17)_=0.6535, *p* = 0.6630; Fig. 5a). In contrast, SW1990, a pancreatic cancer cell line with mutationally activated KRAS but bearing wild type tumour suppressor *P53,* displayed a statistically significant dose-dependent reduction of cell number in response to JVG045 treatment (IC50 = 5.4 μM; One-way ANOVA, F _(5, 12)_=8.208, *p* = 0.0014; Fig. 5a). Western blot analysis on BxPC-3, when probed for pFGFR and pSrc, showed no difference following JVG045 treatment (10 μM) compared to an untreated control in presence or absence of FGF ligand (Fig. 5b). We next determined the effect of JVG045 on cell growth in K-RAS mutated non-pancreatic cancer cell lines. We found that JVG045 is able to inhibit both K-RAS mutated ovarian (OVCAR5) and lung cancer cell lines (A549) in a dose-dependent manner (Supplementary Fig. 5 and Supplementary Table 2).

We also verified the efficacy of JVG045 in a population of pancreatic cancer tumorspheres enriched in cancer stem-like cells [26]. Cancer stem-like cells are slow-cycling cells with a capacity for self-renewal that can elude most therapeutic treatments and are thus responsible for chemoresistance, tumour relapse and metastatic spread to distant sites [45]. We previously showed that pancreatic cancer tumorspheres are highly resistant to the main therapeutic drugs Gemcitabine and Carboplatin [26]. To test its potential as a therapeutic agent, we tested JVG045 on tumorspheres isolated from the human pancreatic cancer cell line AsPC-1. These experiments showed that JVG045 at 10 μM significantly reduced the number of cancer stem-like cells in AsPC-1 (two-sample t-test, t _(6)_  =  9.429; *p* < 0.0001; Fig. 5c). In contrast, when tested on tumorspheres isolated from the KRAS wild type pancreatic cancer cell line BxPC-3, no significant effect of JVG45 was observed (two-sample t-test, t _(4)_  =  1.224; *p* = 0.2880; Fig. 5d). Importantly, primary KPC tumorspheres enriched in cancer stem cells and isolated from primary pancreatic ductal adenocarcinomas in KPC mice showed a significant reduction in cell number after treatment with 10 μM JVG045 (two-sample t-test, t _(4)_  =  4.158; *p* = 0.0142; Fig. 5e) .

### JVG045 reduces in vivo PDAC progression in xenografts models

**Fig. 6:**
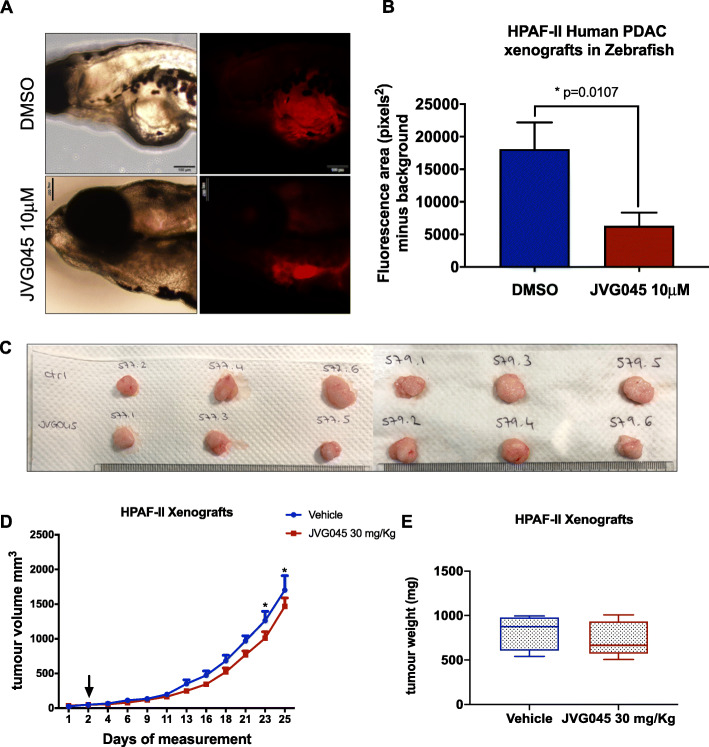
*Effect of JVG045 on zebrafish and mouse xenografts.* **a** Brightfield images and images showing DiL-labelled HPAF-II tumour xenografts in 5 days post-fertilisation zebrafish embryos untreated (DMSO) or treated with JVG045 (10 μM) for 3 days after injection of HPAF-II human pancreatic cancer cells. Scale bar 100 μm. **b** Graph showing the effect of 10 μM JVG045 in reducing the tumour burden in zebrafish embryos (*n* = 12), compared to control embryos (DMSO, *n* = 6). **c** Representative images of HPAF-II tumours from mouse xenografts receiving control treatment (vehicle) or treated with JVG045 (30mh/kg) for 25 days. **d** Graph showing tumour growth of HPAF-II xenograft mouse models treated with vehicle (*n* = 8) and 30 mg/kg JVG045 (*n* = 9). **e** Graph showing the tumour weight at the end of the experiment of HPAF-II xenograft mouse models treated with vehicle (*n* = 8) and 30 mg/kg JVG045 (*n* = 9). Results are expressed as Mean ± SEM

We next tested the antitumor activity of JVG045 in vivo using different xenografts models. Following a previously described protocol [32], we injected 100–200 DiL-labelled (Vybrant™ DiI Cell-Labeling Solution, ThermoFisher Scientific) HPAF-II human pancreatic cancer cells into the perivitelline space of 24 h-old zebrafish embryos. After 24 h, embryos were treated with either DMSO or 10 μM JVG045 for 3 days, until the embryos reached 5 days post fertilisation (Fig. 6a). At the end of the experiment, 15/25 embryos DMSO-treated were alive, while all zebrafish treated with JVG045 survived till the end of the experiment (21/21). Moreover, treatment with the tricarbonyl rhenium compound significantly reduced the overall tumour burden (two-sample t-test, t _(16)_  =  2.887; *p* = 0.0107; Fig. 6b).

To extend these data to a mammalian context, we examined the therapeutic effect of JVG045 on mice harbouring HPAF-II human pancreatic cancer cell line xenografts. These mice were treated with a daily intra-peritoneal injection of JVG045 (30 mg/kg) over a 25-day period; the experiment was terminated when the tumours reached the critical limit volume of 1500mm^3^. Body weight and tumour measurements were recorded to assess whether JVG045 was well tolerated. Data from multiple t-tests for different measurement points showed no significant difference between the weight of xenograft-bearing mice treated with vehicle, compare to those treated with JVG045 (Supplementary Fig. 6). Notably, however, JVG045 was effective in reducing the tumour burden, with a small but significant effect in reducing the tumour volume after 23 days of treatment (mean Vehicle = 1262.483mm^3^ ± 328.433, *n* = 8; mean JVG045 = 1010.422mm^3^ ± 275.295, *n* = 9; t ratio_(158)_ = 2.481; *p* = 0.0141) and after 25 days of treatment (mean Vehicle = 1702.1mm^3^ ± 588.412, *n =* 8; mean JVG045 = 1468.022mm^3^ ± 364.188, *n =* 9; t ratio_(158)_ = 2.499; *p* = 0.0136) (Fig. 6c, d). However, no significant difference was found in the weight of the tumours at the end of the experiment (mean Vehicle = 821 mg ± 187.1, *n =* 8; mean JVG045 = 733.6 mg ± 189.5, *n =* 9; t _(15)_= 0.9580; *p* = 0.3533; Fig. 6e).

**Fig. 7:**
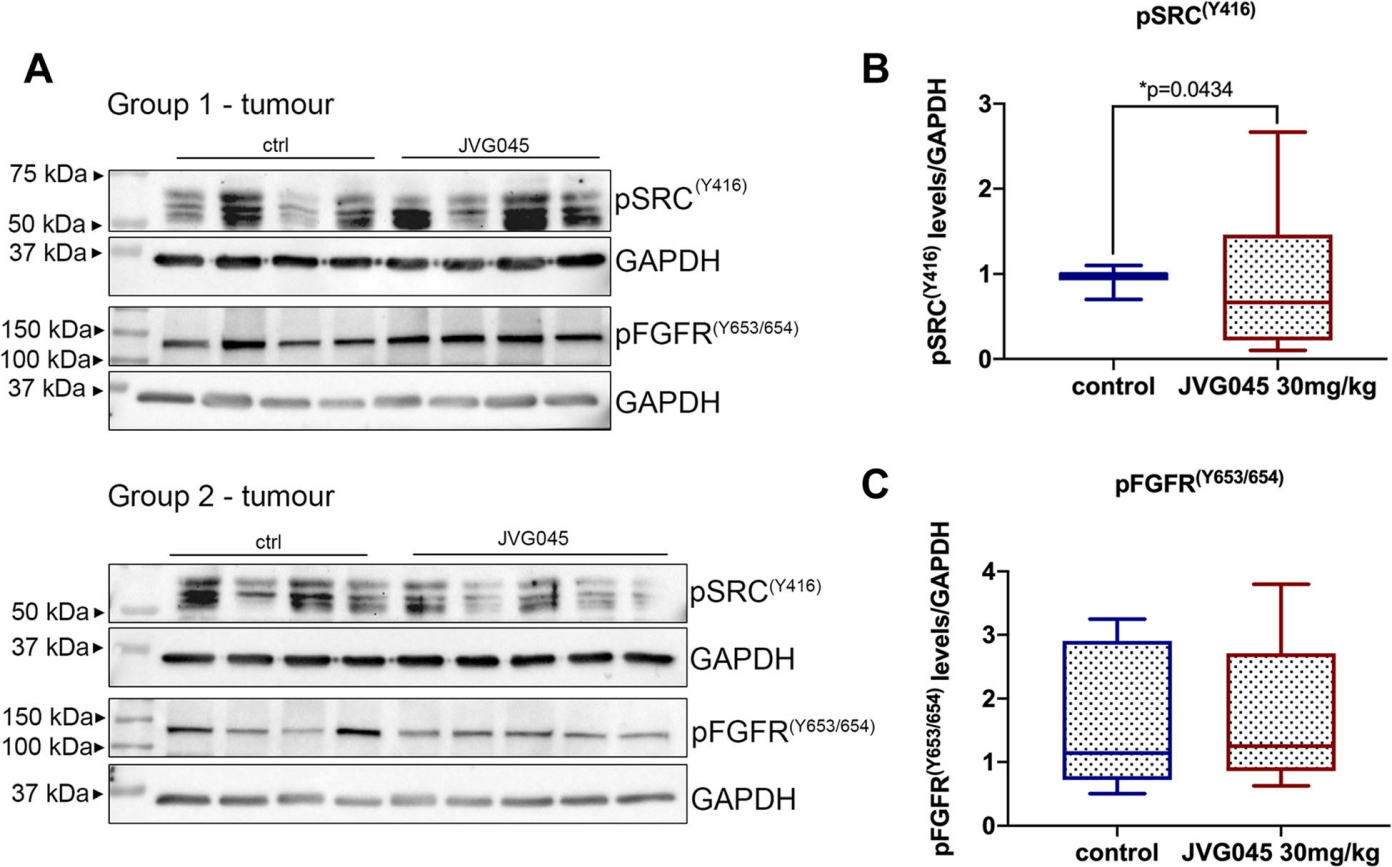
*Effect of JVG045 on FGFR1 and Src in HPAF-II mouse xenografts.* **a** Representative western blot of two groups of tumours either vehicle treated (ctrl) or treated with JVG045 (30 mg/kg) and probed for pSrc (Y416) or pFGFR1(Y653/654). GAPDH was used as loading control. Averaged quantification of (**b**) pSrc(Y416) and (**c**) pFGFR1(Y653/654) western blot signals, normalised to their loading control in vehicle treated tumour xenografts (control) or tumour xenografts treated with JVG045 (30 mg/kg)

To establish whether there was any effect of JVG045 on the phosphorylation of Src at tyrosine residue 416 and/or FGFR or at tyrosine residues 653/654, we prepared extracts from a section of the tumours and performed western blot analyses (Fig. 7a). Quantification of the WB signal was normalised to the loading control and the average data for untreated (control) tumour-bearing mice was compared to the average for tumour-bearing mice treated with JVG045. On average, we observed a significant inhibition of phosphorylation of Src at Tyr416 (Fig. 7b) in animals harbouring tumours treated with JVG045 compared to control (vehicle) treated tumours (two-samples t-test on normalised data t_(14.97)_ = 2.207, *p* = 0.0434). However, no significant difference was detected on the levels of Tyr653/654 phosphorylation of FGFR (two-samples t-test on t _(15)_= 0.04187, *p* = 0.9672; Fig. 7c).

### Physiochemical and metabolic evaluation of JVG045

Finally, we assessed JVG045 for its physiochemical (Table 2) and metabolic properties (Table 2), including kinetic solubility, chromatographic LogD (gLogD) and microsomal stability in human and mouse liver microsomes. JVG045 exhibited poor solubility in pH 6.5 buffer, which remained unchanged in a pH 2 buffer, inferring neutral character. The lipophilic butyl chain is a likely contributor towards the poor solubility of this compound. JVG045 exhibited moderate lipophilicity (Table 2). Metabolic stability was assessed at a substrate concentration of 0.5 μM in human and mouse liver microsomes. JVG045 showed a moderate rate of NADPH-dependent degradation in both human and mouse liver microsomes, with no indication of non-NADPH dependent metabolism in microsomal control samples.

**Table 2:**
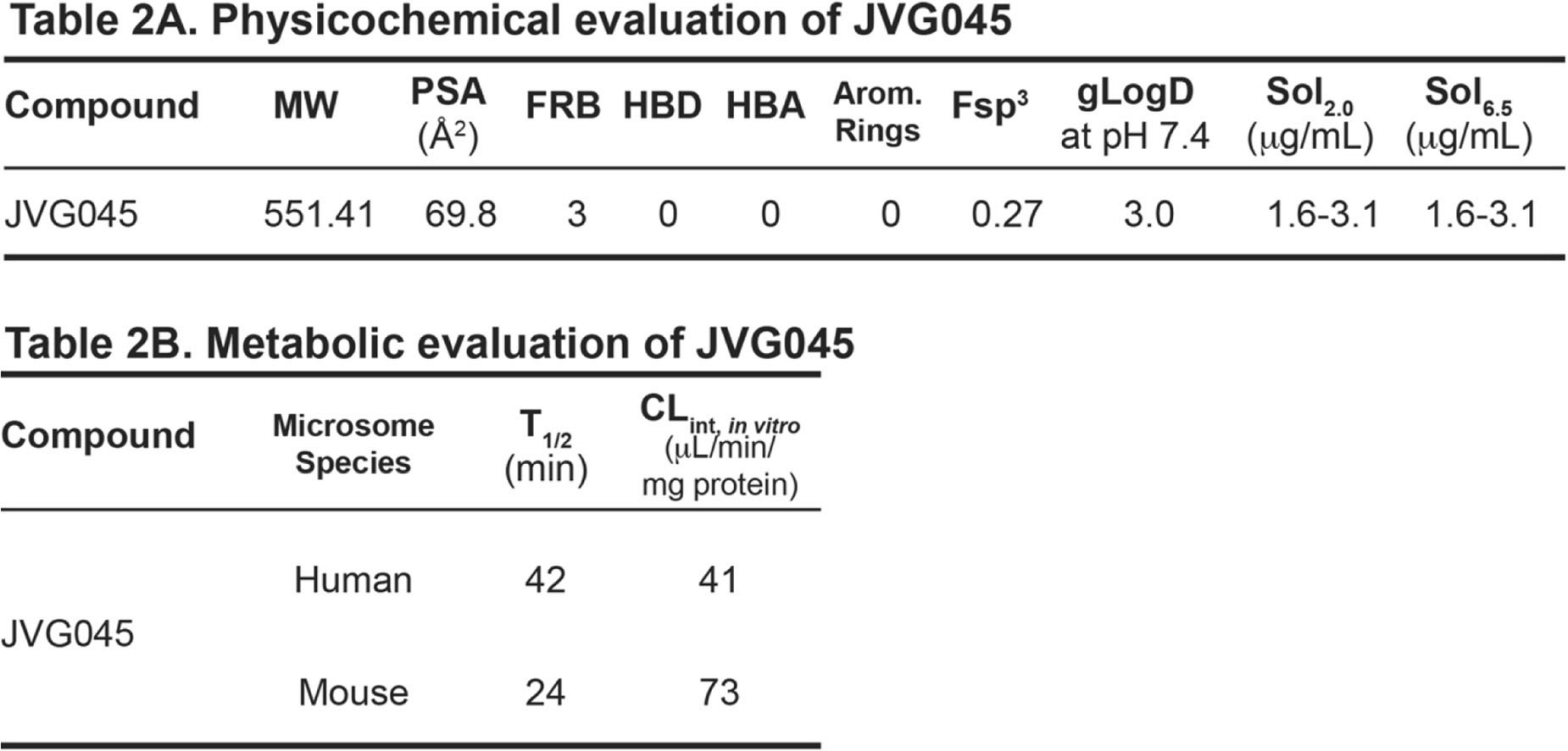
(A) *Physicochemical and* (B) *Metabolic evaluation of JVG045*

## Discussion

Despite the extensive clinical utilization of platinum-based drugs as anticancer agents over more than four decades, their use is currently limited by the occurrence of severe adverse events and the development of chemoresistance. Attempts to substitute platinum with other metals has not produced tangible results in a clinical setting. Among all metal-based drugs, those containing rhenium have recently attracted major interest: several rhenium-based compounds have been tested for their anticancer activity in vitro and in vivo with promising results [3, 8, 9, 24, 46]. However, the lack of a known mechanism of action and molecular target represents a major obstacle in advancing these therapeutics to the clinical trial stage. In previous work, we identified Re-NHC complexes that can induce G2/M arrest and inhibition of the aurora kinase A phosphorylation [25]. Here, we characterized the molecular targets of our leading compounds and evaluated their anticancer potential in vitro and in vivo. Based on the data we present, we surmise that our rhenium-based compounds target FGFR and Src, possibly through ROS interaction with specific cysteine residues. How exactly the rhenium compounds inhibit FGFR1 and Src remains to be determined. It is currently unclear why our compounds showed inhibitory effects in the kinases screen but not in the individual kinase activity or autophosphorylation assay. A possible explanation is the presence of different amounts of redox reagents such as dithiothreitol. Our proposed ROS-based mechanism is consistent with previous work showing that both FGFR and Src are regulated by ROS-mediated cysteine oxidation [36]. On the other hand, our previous work suggested that low levels of ROS can enhance Src activation, and therefore JVG045, by targeting the conserved cysteine residue, could prevent such oxidative activation [30]. Another aspect that needs to be determined is the lack of embryonic teratogenicity of JVG045 compared to the pan-FGFR kinase inhibitor BGJ398. Zebrafish embryo development is dependent on FGFRs and the four FGFRs present in zebrafish are highly redundant; it is possible that JVG045 is not able to block all zebrafish FGFRs [42]. Alternatively, a possible explanation could be that the targeted cysteine is not present in the zebrafish FGFR. Indeed, a comparison of the human FGFRs sequences shows that in the zebrafish counterpart few cysteine residues are missing [47–49]. Interestingly, recent data demonstrated that aurora kinase A is also regulated by reversible cysteine oxidation, suggesting that our observed Aurora Kinase inhibition by JVG045 [25] could occur through a similar proposed mechanism [50]. Our results showed that JVG045 is rather specific in targeting K-Ras mutated pancreatic cancer cells and MYCN amplified neuroblastoma cells. The toxicity profile of our lead compound in zebrafish and mouse is also very favourable. The anticancer activity of our lead compound was also investigated using zebrafish and mice models. Our results showed that JVG045 is able to inhibit tumour growth in vivo, although the activity in mice was less impressive compared to the zebrafish model. This is likely due to solubility issues as shown by our physiochemical studies. Furthermore, we could confirm in vivo inhibition of Src phosphorylation but not FGFR1. This indicates that JVG045 is able to reach the tumour but probably the concentration and/or the duration of persistence in the circulation is suboptimal. Although our study provides strong evidence of in vivo activity for rhenium tricarbonyl compounds, it is clear that the pharmacodynamics and pharmacokinetics of these compounds require improvement. Moreover, it would be interesting to see whether multiple administrations (i.e. twice a day) or higher concentrations are able to increase the anti-tumour activity in mouse models.

## Conclusions

In conclusion, this work provides novel mechanistic insight and the molecular targets for Re-NHC complexes and demonstrates their specificity in blocking cancer growth in vitro and in vivo. The minimal or lack of activity of these compounds in non-malignant cells and in cancer cells with wild type KRas and low levels of MYCN is a promising feature for the development of novel therapies. The identification of the mechanism of action and of the molecular targets is pivotal for the advancement of these agents in an effort to develop efficacious and non-toxic therapeutics. Both FGFR1 and Src play a key role in cancer progression and it is therefore anticipated that the development of these novel anticancer agents could have a broad spectrum of application in different cancer settings.

## Supplementary Information

**Additional file 1: Supplementary Figures: 1–6; Supplementary Tables: 1–2**.

## Data Availability

The data that support the findings of this study are available from the corresponding author upon reasonable request.

## Abbreviations

FGFR: Fibroblast Growth Factor Receptor
GAPDH: Glyceraldehyde-3-phosphate dehydrogenase
KPC: KrasLSL.G12D/+; p53R172H/+; PdxCretg/+
Re-NHC: Rhenium N-heterocyclic carbene
ROS: Reactive oxygen species
