# Supplementary material for: Rhenium N-heterocyclic carbene complexes block growth of aggressive cancers by inhibiting FGFR- and SRC-mediated signalling
Source: J Exp Clin Cancer Res. 2020 Dec 7;39:276. doi: 10.1186/s13046-020-01777-7 (PMC7720599; doi:10.1186/s13046-020-01777-7)
Supplement: Supplementary file 1 — Additional file 1: Supplementary Figures: 1–6; Supplementary Tables: 1–2. [file 13046_2020_1777_MOESM1_ESM.docx]

**Supplementary Material**

**
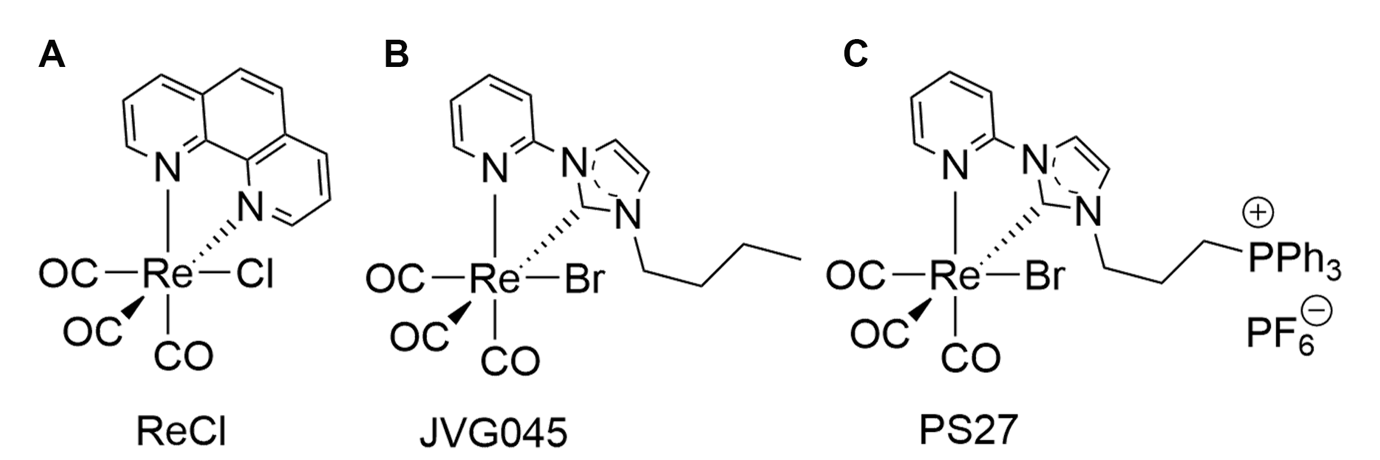
**

**Supplementary Figure 1:** Chemical structures of [Re(CO)3(phen)Cl] (A) referred to as ReCl; JVG045 (B) and ps27 (C).

**
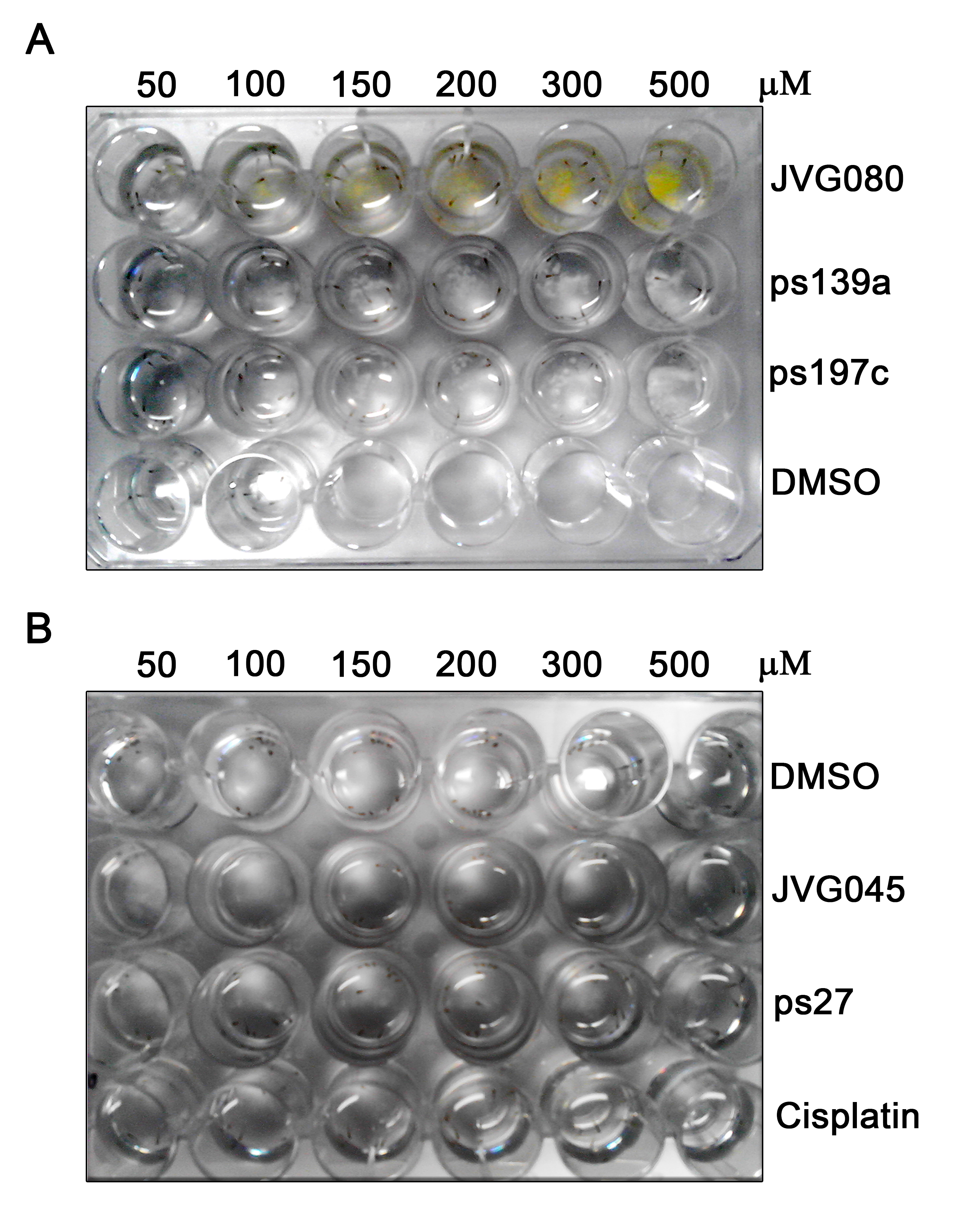
**

**Supplementary Figure 2:** Pictures showing solubility of selected Rhenium compounds compared to DMSO and Cisplatin after 72h of incubation.


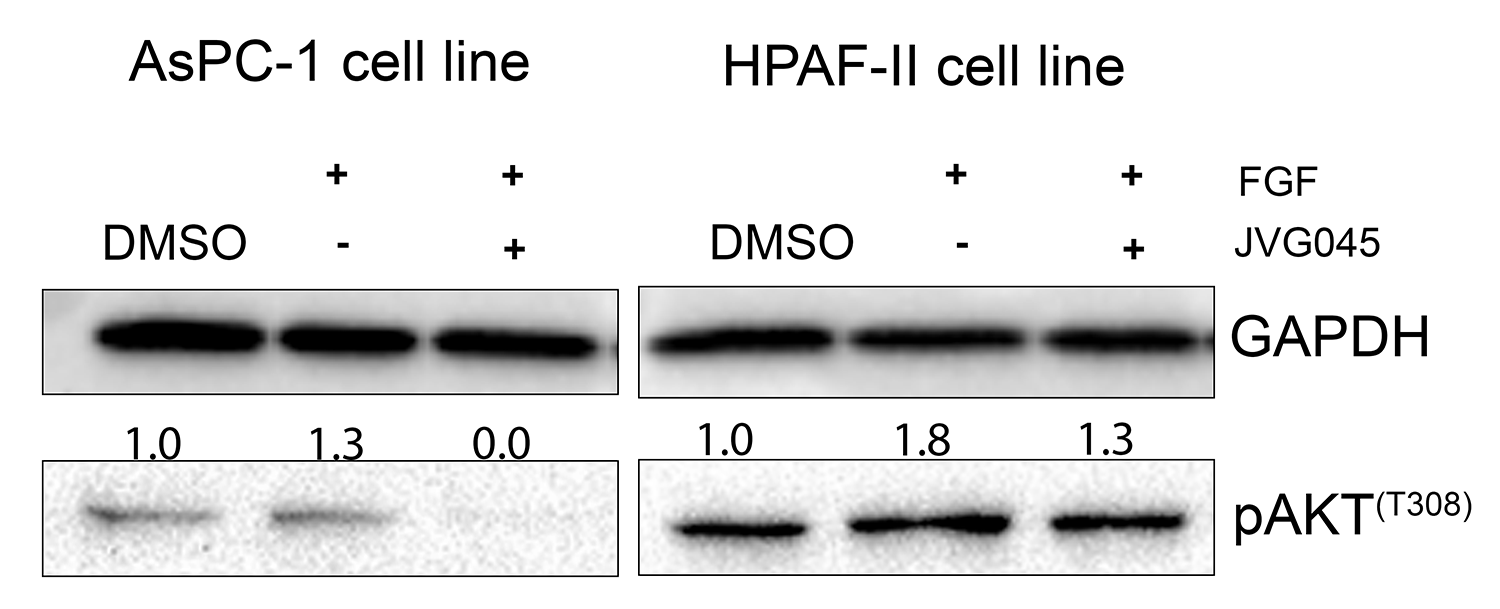


**Supplementary Figure 3:** Representative western blot (of three independent experiments performed) showing the inhibitory effect of JVG045 (10µM, 30’ pre-treatment) toward the FGF (20 ng/ml)-induced phosphorylation of Akt Thr 308.


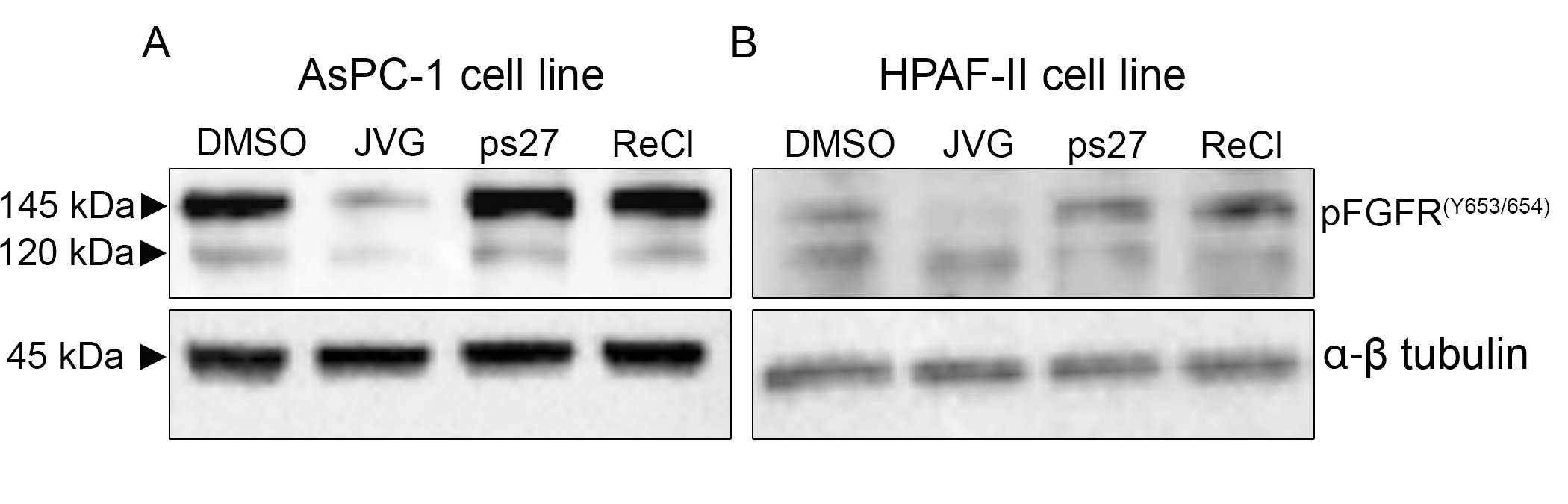
**Supplementary Figure 4:** Representative western blot (of three independent experiments performed) comparing the inhibitory effect of the two rhenium compounds JVG045 to ps27 (both used at 10µM) toward the phosphorylation of FGFR. ReCl was used as a negative control.


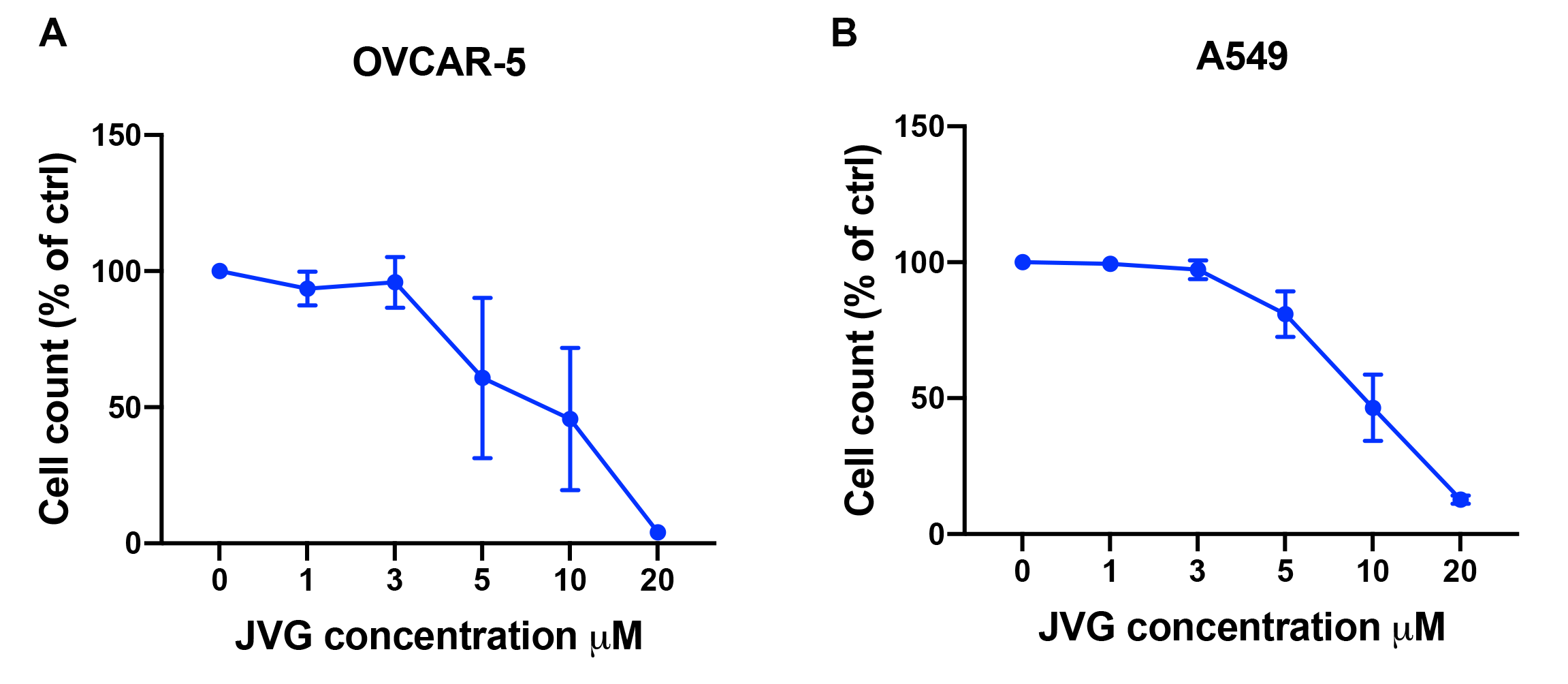


**Supplementary Figure 5:** Dose response of JVG045 on human ovarian (OVCAR5) and lung (A549) K-Ras mutated cancer cell lines. (A) Graph showing that in the OVCAR-5 human ovarian cancer and (B) in the A549 human lung cancer cell lines, both bearing a *KRAS* mutation, increasing doses of JVG045 had a significant effect on cell number.

Experiments (OVCAR5 n=3; A549 n=4) were performed in triplicate and showed as Mean ±SEM.


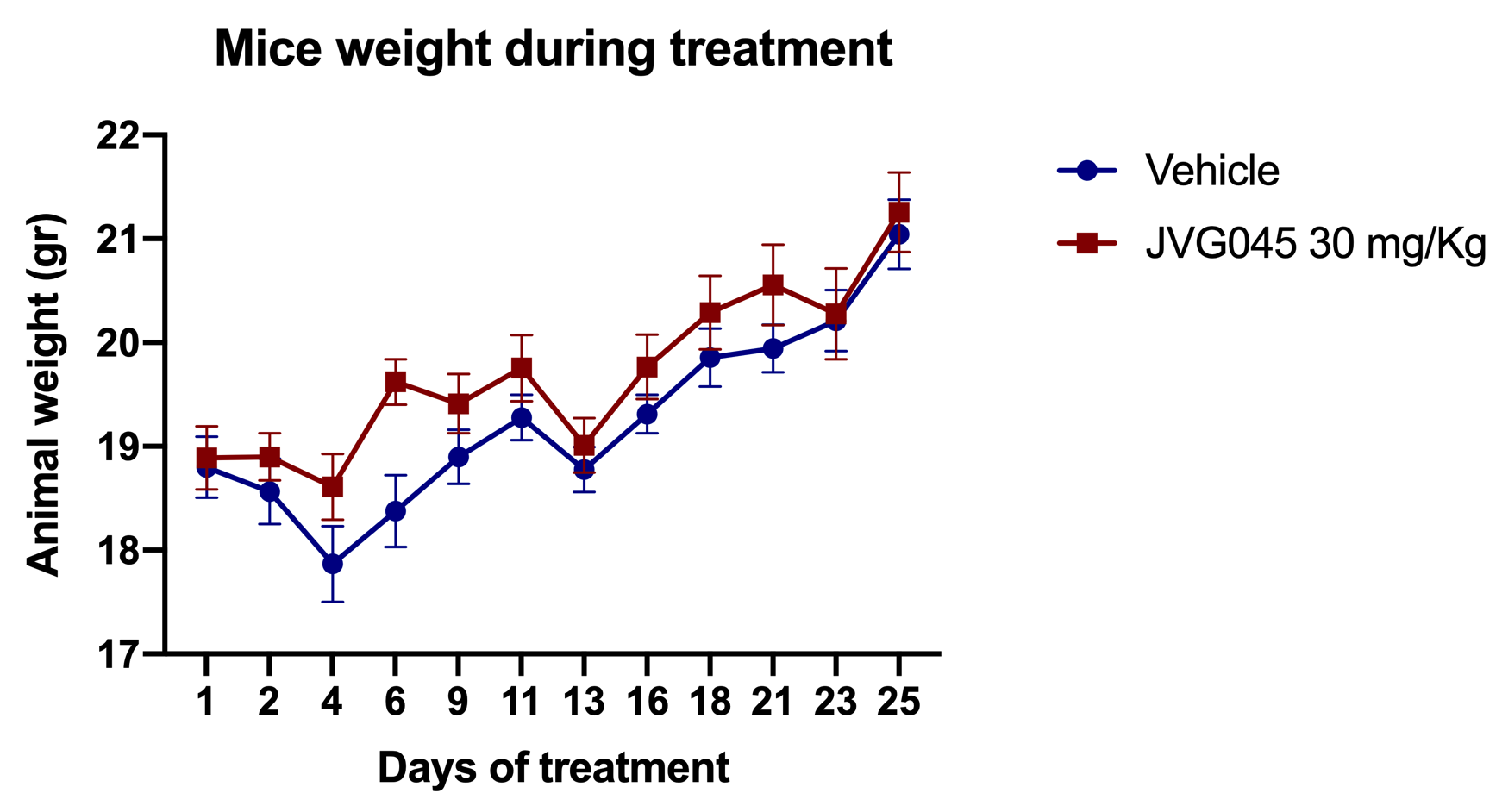


**Supplementary Figure 6:** Graph showing weight changes in xenograft-bearing mice treated with vehicle or with JVG045 (30mg/kg) for the duration of treatment.

|  | JVG045 (1000nM) | Ps 27  (1000nM) |
| --- | --- | --- |
| AKT1 (PKB alpha) - ZLYTE - Km app | -1 | -5 |
| AKT2 (PKB beta) - ZLYTE - Km app | -11 |  |
| AKT3 (PKB gamma) - ZLYTE - Km app | 7 | 10 |
| ALK - ZLYTE - Km app | -13 | -15 |
| AMPK A1/B1/G1 - ZLYTE - Km app | 17 | 17 |
| AMPK A2/B1/G1 - ZLYTE - Km app | 30 |  |
| AURKA (Aurora A) - ZLYTE - Km app | 8 | 7 |
| AURKC (Aurora C) - ZLYTE - Km app | 9 | 12 |
| AXL - ZLYTE - Km app | -4 | -4 |
| BLK - ZLYTE - Km app | 9 | 9 |
| BMPR2 - LanthaScreen Binding - Eu-anti-His | 8 |  |
| BMX - ZLYTE - Km app | 23 | 24 |
| BRAF - LanthaScreen Binding - Eu-anti-GST | -1 | 1 |
| BTK - ZLYTE - Km app | 14 | 13 |
| CAMK1 (CaMK1) - Adapta - 10 | 0 | 13 |
| CAMK2A (CaMKII alpha) - ZLYTE - Km app | 6 | 2 |
| CDC42 BPA (MRCKA) - ZLYTE - Km app | 2 | 2 |
| CDK1/cyclin B - ZLYTE - Km app | -2 | -6 |
| CDK2/cyclin A1 - LanthaScreen Binding - Eu-anti-GST | -9 | 4 |
| CDK4/cyclin D1 - Adapta - 10 | 15 | 18 |
| CDK5/p25 - ZLYTE - Km app | 10 | 11 |
| CDK5/p35 - ZLYTE - Km app | 3 | 0 |
| CDK6/cyclin D1 - Adapta - 10 | 10 | 9 |
| CHEK1 (CHK1) - ZLYTE - Km app | 13 | 14 |
| CHEK2 (CHK2) - ZLYTE - Km app | 13 | 11 |
| CHUK (IKK alpha) - Adapta - Km app | -4 | -4 |
| CLK1 - ZLYTE - Km app | 2 | 4 |
| DDR1 - LanthaScreen Binding - Eu-anti-GST | 2 | 10 |
| DDR2 - LanthaScreen Binding - Eu-anti-GST | 11 | 8 |
| EGFR (ErbB1) - ZLYTE - Km app | -17 | -18 |
| EPHA1 - ZLYTE - Km app | -1 | -7 |
| ERBB2 (HER2) - ZLYTE - Km app | -7 | -6 |
| FGFR1 - ZLYTE - Km app | 56 | 59 |
| FRAP1 (mTOR) - ZLYTE - Km app | -14 | -13 |
| FYN - ZLYTE - Km app | -7 | -8 |
| GRK5 - ZLYTE - Km app | -2 | -2 |
| GSK3A (GSK3 alpha) - ZLYTE - Km app | 6 | 7 |
| GSK3B (GSK3 beta) - ZLYTE - Km app | 12 | 12 |
| IGF1R - ZLYTE - Km app | 7 | 5 |
| KDR (VEGFR2) - ZLYTE - Km app | 4 | 4 |
| KIT - ZLYTE - Km app | 0 | 0 |
| LIMK2 - LanthaScreen Binding - Eu-anti-GST | 4 |  |
| MAP2K1 (MEK1) - LanthaScreen Binding - Eu-anti-His | 1 | -6 |
| MAP2K2 (MEK2) - LanthaScreen Binding - Eu-anti-His | -7 | -1 |
| MAP3K10 (MLK2) - LanthaScreen Binding - Eu-anti-GST | 5 |  |
| MAPK1 (ERK2) - ZLYTE - Km app | -8 | -7 |
| MAPK14 (p38 alpha) Direct - ZLYTE - Km app | 15 | 17 |
| MAPK3 (ERK1) - ZLYTE - Km app | 1 | 0 |
| MAPK8 (JNK1) - LanthaScreen Binding - Eu-anti-His | 17 | 21 |
| MAPK9 (JNK2) - LanthaScreen Binding - Eu-anti-His | 12 | 6 |
| MAPKAPK2 - ZLYTE - Km app | 10 | 16 |
| MARK1 (MARK) - ZLYTE - Km app | 1 | 2 |
| MET (cMet) - ZLYTE - Km app | 23 | 29 |
| PAK1 - ZLYTE - Km app | 9 | 10 |
| PAK6 - ZLYTE - Km app | 3 | 3 |
| PDGFRA (PDGFR alpha) - ZLYTE - Km app | -7 | -7 |
| PDK1 Direct - ZLYTE - Km app | 1 | -1 |
| PI4K2A (PI4K2 alpha) - Adapta - Km app | -5 | -4 |
| PI4K2B (PI4K2 beta) - Adapta - Km app | -9 |  |
| PI4KA (PI4K alpha) - Adapta - 10 | 2 | 6 |
| PI4KB (PI4K beta) - Adapta - Km app | -2 | 5 |
| PIK3C2A (PI3K-C2 alpha) - Adapta - Km app | -6 | -5 |
| PIK3C2G (PI3K-C2 gamma) - Adapta - Km app | -8 | -7 |
| PIK3C3 (hVPS34) - Adapta - Km app | -3 | -5 |
| PIK3CB/PIK3R1 (p110 beta/p85 alpha) - Adapta - Km app | 2 | 2 |
| PIK3CD/PIK3R1 (p110 delta/p85 alpha) - Adapta - Km app | 10 | 14 |
| PIK3CG (p110 gamma) - Adapta - Km app | 9 | 14 |
| PIP5K1A - Adapta – 10 | 3 | -1 |
| PIP5K1B - Adapta – 10 | 2 | 3 |
| PLK1 - ZLYTE - Km app | 11 | 12 |
| PRKACA (PKA) - ZLYTE - Km app | 1 | 0 |
| RET - ZLYTE - Km app | -3 | -5 |
| ROCK1 - ZLYTE - Km app | 4 | 6 |
| ROCK2 - ZLYTE - Km app | 9 | 10 |
| ROS1 - ZLYTE - Km app | 0 | -4 |
| RPS6KA1 (RSK1) - ZLYTE - Km app | 6 | 4 |
| RPS6KB1 (p70S6K) - ZLYTE - Km app | 2 | -1 |
| SGK (SGK1) - ZLYTE - Km app | 31 | 32 |
| SGK2 - ZLYTE - Km app | 22 | 22 |
| SGKL (SGK3) - ZLYTE - Km app | 26 | 30 |
| SPHK1 - Adapta - Km app | 33 | 35 |
| SRC - ZLYTE - Km app | 55 | 54 |
| STK32B (YANK2) - LanthaScreen Binding - Eu-anti-GST | 6 |  |
| STK39 (STLK3) - LanthaScreen Binding - Eu-anti-His | -4 |  |
| SYK - ZLYTE - Km app | 5 | 5 |
| ULK1 - LanthaScreen Binding - Eu-anti-GST | 2 |  |
| YES1 - ZLYTE - Km app | 21 | 20 |
| ABL1 - ZLYTE - Km app | -25 | -25 |
| AKT2 (PKB beta) - ZLYTE - Km app |  | -29 |
| AMPK A2/B1/G1 - ZLYTE - Km app |  | 19 |
| AURKB (Aurora B) - ZLYTE - Km app | 9 | 13 |
| CSNK1A1 (CK1 alpha 1) - ZLYTE - Km app | -38 | -27 |
| CSNK1G1 (CK1 gamma 1) - ZLYTE - Km app | -53 | -55 |
| CSNK2A2 (CK2 alpha 2) - ZLYTE - Km app | -41 | -44 |
| FLT1 (VEGFR1) - ZLYTE - Km app | -34 | -42 |
| INSR - ZLYTE - Km app | -2 | -7 |
| IRAK4 - ZLYTE - Km app | -23 | -36 |
| ITK - ZLYTE - Km app | -27 | -21 |
| JAK1 - ZLYTE - Km app | 0 | -1 |
| PRKCA (PKC alpha) - ZLYTE - Km app | 7 | 8 |
| PRKCB1 (PKC beta I) - ZLYTE - Km app | -2 | -1 |
| PRKCD (PKC delta) - ZLYTE - Km app | 0 | 2 |
| PRKCE (PKC epsilon) - ZLYTE - Km app | 9 | 10 |
| PRKCG (PKC gamma) - ZLYTE - Km app | 5 | 2 |
| PRKCZ (PKC zeta) - ZLYTE - Km app | 4 | 5 |
| PI4K2B (PI4K2 beta) - Adapta - Km app | -9 |  |
| PIK3C2B (PI3K-C2 beta) - Adapta - 10 | 8 | 8 |
| PIK3CA/PIK3R1 (p110 alpha/p85 alpha) - Adapta - Km app | 9 | -6 |
| PIP4K2A - Adapta – 10 |  | -14 |
| PIP5K1C - Adapta – 10 | -15 | -10 |
| PIP4K2A - Adapta – 10 | -21 |  |
| NEK2 - ZLYTE - Km app |  | 3 |
| PAK4 - ZLYTE - Km app |  | -5 |
| PIM2 - ZLYTE - Km app |  | 3 |
| PTK2 (FAK) - ZLYTE - Km app |  | 8 |
| PTK2B (FAK2) - ZLYTE - Km app |  | 6 |

**Supplementary Table 1:** Summary of results from SelectScreen Kinase Profiling. JVG045 and ps27 show a >50% inhibitory activity towards SRC and FGFR1.


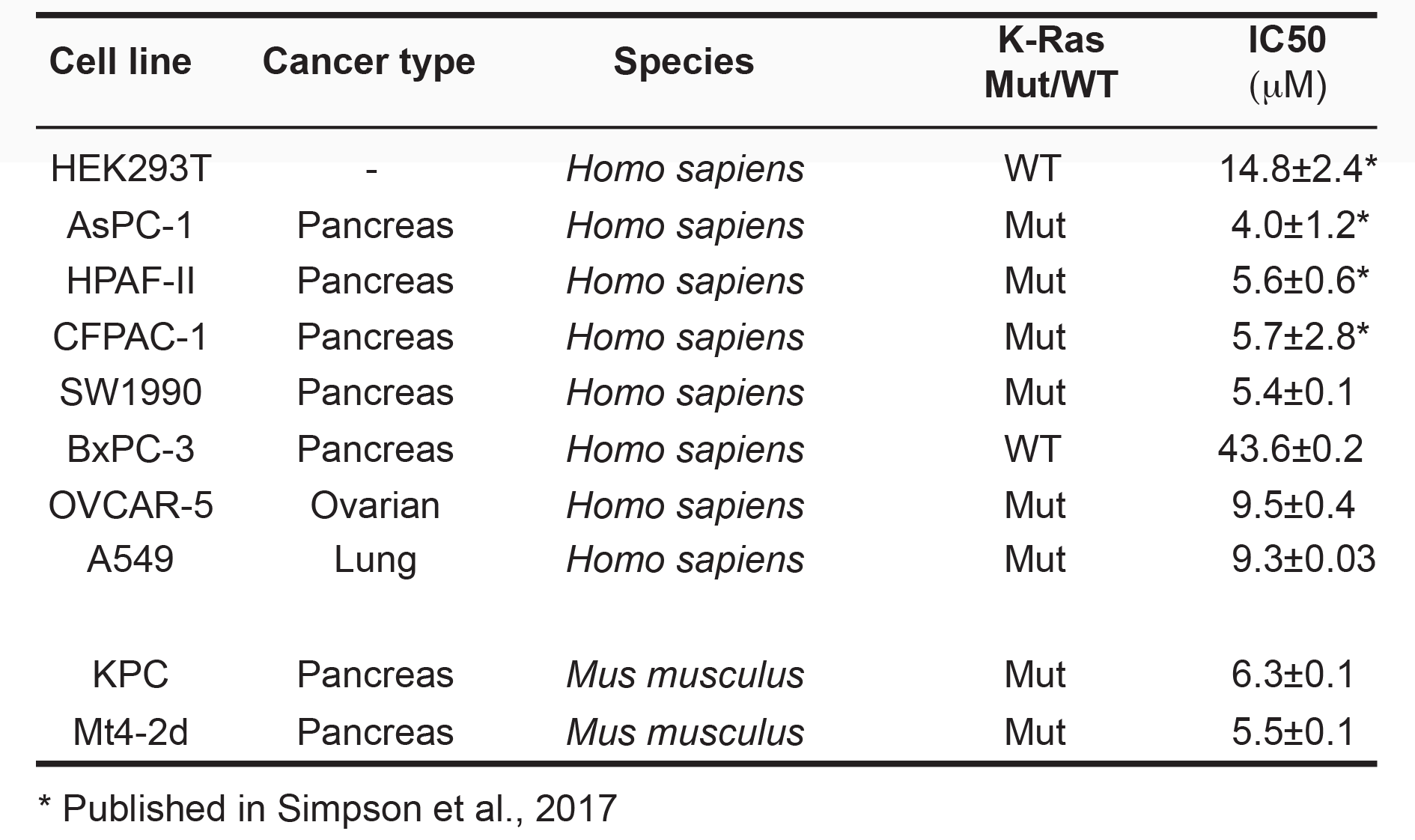


**Supplementary Table 2:** JVG045 IC50 values for different human and murine K-Ras mutated and wild-type cell lines and non -cancerous HEK293T cell line.
